# Variation in cognitive insight processes between schizophrenia and bipolar disorder in a Tunisian population

**DOI:** 10.1192/j.eurpsy.2022.2023

**Published:** 2022-09-01

**Authors:** M. Ben Hmida

**Affiliations:** Université de Tunis, Faculté des sciences humaines et sociales de Tunis, Psychology, tunis, Tunisia

**Keywords:** schizophrénia, cognitive insight, Tunisian population, bipolar disorder

## Abstract

**Introduction:**

Cognitive insight is a relatively recent concept referring to the ability, not only to reassess unusual experiences objectively after corrective feedback but also to distance oneself from them and it seems to be specifically altered in schizophrenia. Yet, despite its importance in the understanding of psychotic symptoms, this process has never been studied in the North African population.

**Objectives:**

Therefore, this paper aims to compare cognitive insight performances between two Tunisian psychiatric populations and to explore its relationship with other cognitive processes.

**Methods:**

The study population comprised 17 participants with schizophrenia, 9 with bipolar disorder, and 30 healthy controls. The groups were paired for age, education level, and socioeconomic status. We assessed depression, global executive functioning, verbal episodic memory, metamemory (online and offline), and cognitive insight. The latter was evaluated by the Beck Cognitive Insight Scale.

**Results:**

The results showed that, compared to the other groups, participants with schizophrenia obtained a lower self-reflectiveness score and a higher self-certainty score, resulting in a significantly lower composite index. These findings seem to indicate the alteration of cognitive insight in schizophrenia. However, no significant differences were found between the other two groups. Moreover, correlational analyses showed that cognitive insight components were only associated with metamemory indices which proved to be the best predictors of this ability, along with the global executive score.

**Conclusions:**

In conclusion, our data seems to corroborate the international literature reporting a cognitive insight deficit in schizophrenia. However, further research is needed in order to better understand the specific processes underlying this metacognitive function.

**Disclosure:**

No significant relationships.

